# *Caenorhabditis elegans-*Based *Aspergillus fumigatus* Infection Model for Evaluating Pathogenicity and Drug Efficacy

**DOI:** 10.3389/fcimb.2020.00320

**Published:** 2020-06-26

**Authors:** Chukwuemeka Samson Ahamefule, Qijian Qin, Arome Solomon Odiba, Siqiao Li, Anene N. Moneke, James C. Ogbonna, Cheng Jin, Bin Wang, Wenxia Fang

**Affiliations:** ^1^National Engineering Research Center for Non-food Biorefinery, Guangxi Academy of Sciences, Nanning, China; ^2^College of Life Science and Technology, Guangxi University, Nanning, China; ^3^Department of Microbiology, University of Nigeria, Nsukka, Nigeria; ^4^State Key Laboratory of Non-food Biomass and Enzyme Technology, Guangxi Academy of Sciences, Nanning, China

**Keywords:** *Caenorhabditis elegans*, *Aspergillus fumigatus*, infection model, hyphal filamentation, pathogenicity

## Abstract

*Aspergillus fumigatus* is the most reported causative pathogen associated with the increasing global incidences of aspergilloses, with the health of immunocompromised individuals mostly at risk. Monitoring the pathogenicity of *A. fumigatus* strains to identify virulence factors and evaluating the efficacy of potent active agents against this fungus in animal models are indispensable in current research effort. *Caenorhabditis elegans* has been successfully utilized as an infection model for bacterial and dimorphic fungal pathogens because of the advantages of being time-efficient, and less costly. However, application of this model to the filamentous fungus *A. fumigatus* is less investigated. In this study, we developed and optimized a stable and reliable *C. elegans* model for *A. fumigatus* infection, and demonstrated the infection process with a fluorescent strain. Virulence results of several mutant strains in our nematode model demonstrated high consistency with the already reported pathogenicity pattern in other models. Furthermore, this *C. elegans-A. fumigatus* infection model was optimized for evaluating the efficacy of current antifungal drugs. Interestingly, the azole drugs in nematode model prevented conidial germination to a higher extent than amphotericin B. Overall, our established *C. elegans* infection model for *A. fumigatus* has potential applications in pathogenicity evaluation, antifungal agents screening, drug efficacy evaluation as well as host-pathogen interaction studies.

## Introduction

*Aspergillus fumigatus* is a saprophytic environmental fungus with ubiquitous airborne spores. It is also an opportunistic fungal pathogen responsible for mycoses including invasive aspergillosis (IA) mostly in immunocompromised patients (Van De Veerdonk et al., 2017; Fang and Latge, 2018). IA is a very severe systemic infection with an estimated global incidence of over 200,000 per annum (Geissel et al., 2018) and a mortality rate close to 100% in most groups of patients who did not receive treatment (Darling and Milder, 2018). Unfortunately, cases of aspergillosis have also been reported in immunocompetent patients (Stevens and Melikian, 2011). The emergence of drug resistant strains to the wide range of currently available drugs has posed a serious challenge as cases are rising globally (Prigitano et al., 2017, 2019; Abdolrasouli et al., 2018). Reports of *A. fumigatus* resistance to azole drugs (Hagiwara et al., 2017; Sharma et al., 2019), polyene drugs such as amphotericin B (Ashu et al., 2018), and echinocandin drugs (Beer et al., 2018) are increasing in both clinical (Prigitano et al., 2017; Abdolrasouli et al., 2018; Hagiwara et al., 2018) and environmental isolates (Vaezi et al., 2018; Prigitano et al., 2019). With the limited repertoire of antifungal drug classes, there is therefore the need to urgently discover new therapeutic option for this infection.

Notable strategy will involve studying key virulence factors in suitable model as a tool for understanding pathogenesis as well as screening and testing the efficacy of antifungal agents. *Caenorhabditis elegans* possesses great advantages such as simple life cycle, ease of cultivation and manipulation, relatively low space as well as no ethical requirement, making it an excellent model for a broad range of pathological diseases and infection. Indeed, *C. elegans* has been widely utilized as a host model for pathogenic bacterial infections, such as *C. elegans-Pseudomonas aeruginosa* (Darby et al., 1999; Tan et al., 1999; Zaborin et al., 2009), *C. elegans-Salmonella* spp. (Labrousse et al., 2000), *C. elegans-Yersinia pestis* (Styer et al., 2005), *C. elegans-Staphylococcus aureus* (Thompson and Brown, 2017), *C. elegans-Streptococcus pyogenes* (Jansen et al., 2002), and *C. elegans-Acinetobacter baumanii* (Vallejo et al., 2015). It has also been adopted for screening of active antimicrobial compounds and discovery of effective agents against several human bacterial (Kong et al., 2014; Tharmalingam et al., 2018) and fungal pathogens (Tampakakis et al., 2008; Okoli et al., 2009). Furthermore, *C. elegans* model has been applied for evaluating the pathogenicity of a number of clinically relevant fungal pathogens, including *Candida albicans, Candida krusei, Candida parapsilosis, Candida glabrata* (Breger et al., 2007), *Histoplasma capsulatum* (Johnson et al., 2009), *Cryptococcus neoformans* (Mylonakis et al., 2002), and *Penicillium marneffei* (Huang et al., 2014).

Currently *Galleria mellonella* (Gomez-Lopez et al., 2014), *Bombyx mori* (Nakamura et al., 2017), *Drosophila melanogaster* (Lionakis and Kontoyiannis, 2012), mice (Paulussen et al., 2015), and guinea pigs (Wiederhold et al., 2015) have been utilized as *in vivo* models for *A. fumigatus* infection. On the contrary, little attention has been given to the application of *C. elegans* model in filamentous fungal infections, except for the only report in *A. fumigatus* (Okoli and Bignell, 2015). Part of the limitations to its use is that the co-culture technique of infecting *C. elegans* is only suitable for bacterial or dimorphic fungal pathogens but not appropriate for filamentous fungi, as rapid conidia germination and intensive hyphal filamentation would interfere with the survival assessment of worms in killing assay. Despite the fact that Okoli and Bignell have demonstrated the possibility of adopting the *C. elegans* model for *A. fumigatus* infection, further optimization is required to increase the efficiency in worms-spores separation and shorten operation time. This will enable suitable applications of the model in high-throughput screening for novel antifungal compounds, evaluating pathogenicity as well as testing the efficacy of current antifungal agents. In this study, we established a stable and reliable *C. elegans-A. fumigatus* infection model, confirmed by evaluating the pathogenicity of several *A. fumigatus* mutants strains. The model was also optimized for evaluating the efficacy of currently used antifungal agents.

## Results

### Establishment and Optimization of *C. elegans* Model for *A. fumigatus* Infection

**Figure 1:**
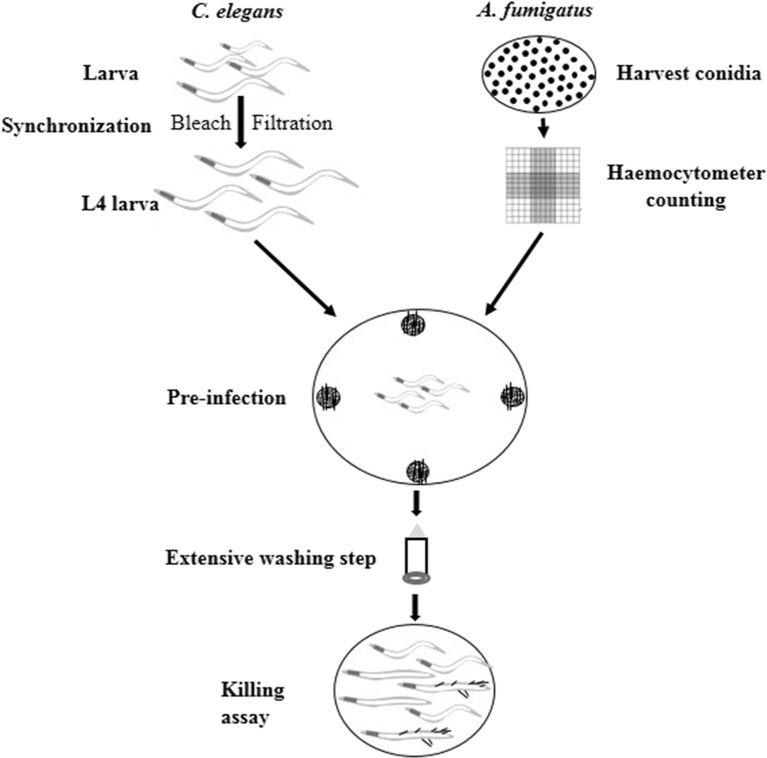
Schematic procedure of *C. elegans*-based *A. fumigatus* infection model. Synchronized L4 stage worms were put into NGM plates containing *A. fumigatus* spores at the four edges for 16 h pre-infection. Then extensive washing step was applied to remove conidia that were not ingested by worms. Finally the washed worms were added to BHI medium for killing assay.

**Figure 2:**
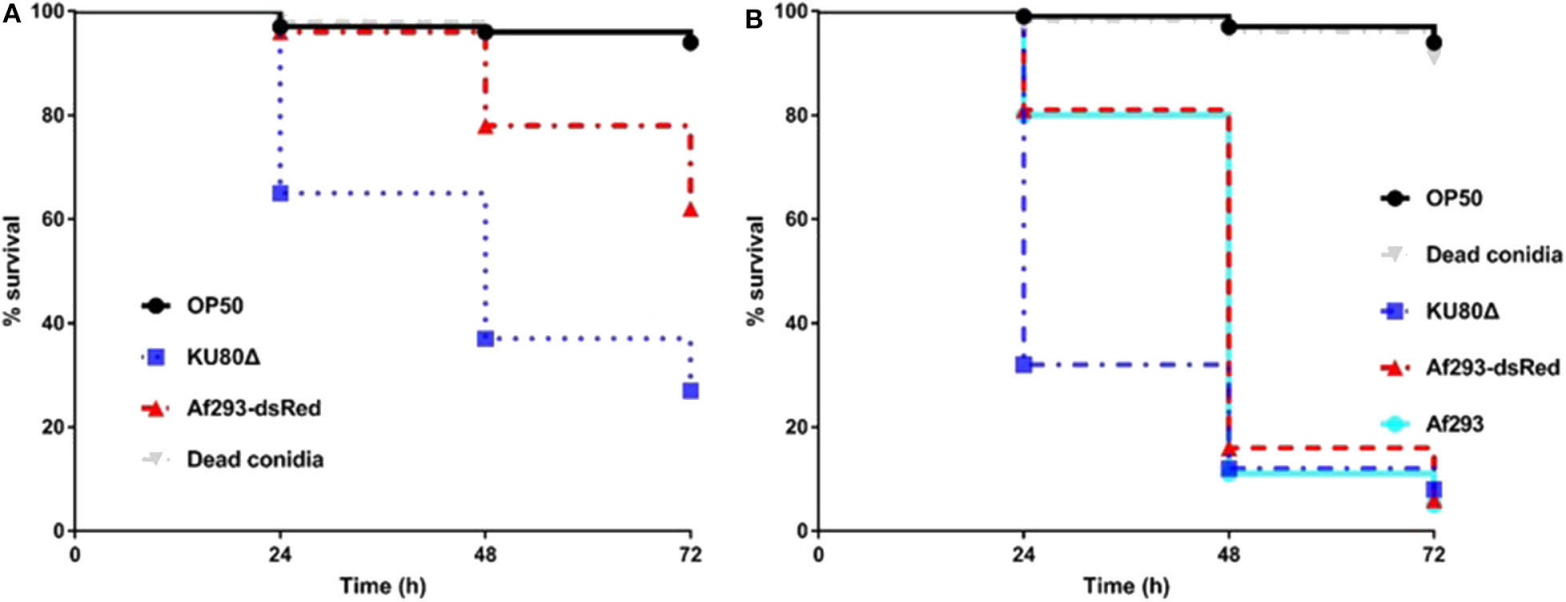
Survival rate comparison in *C. elegans* hosts. **(A)**, Kaplan-Meier survival plots of *fem-3*(q96) fed by *E.coli* OP50 or conidia of indicated *A. fumigatus* strains. **(B)**, Kaplan-Meier survival plots of *glp-4*(bn2); *sek-1*(km4) fed by *E.coli* OP50 or conidia of indicated *A. fumigatus* strains. Compared to OP50 and dead conidia the Af293-dsRed and KU80Δ strains exhibited significant pathogenicity [*P* < 0.0001, Log-rank (Mantel-Cox) test] in both *fem-3*(q96) and *glp-4*(bn2); *sek-1*(km4) worms. Three biological repeats (each with triplicates) were conducted for each strain.

*C. elegans* has been successfully utilized as an infection model for several clinically relevant fungal pathogens, such as *C. albicans, C. glabrata, C. neoformans*, and *H. capsulatum* (Mylonakis et al., 2002; Breger et al., 2007; Johnson et al., 2009), but with limited application in filamentous fungal pathogens like *A. fumigatus*. Considering the fast germination feature of *A. fumigatus* in Brain Heart Infusion medium (BHI), we adopted the pre-infection technique on solid plates as described by Okoli and Bignell (35). An extensive washing technique using hand-made device of filter membrane-attached-on-tube was applied to remove conidia that were not ingested by the worms at pre-infection assay. The pre-infection time, conidia concentration for adequate infection and suitable worm numbers for both pre-infection and post-infection were optimized. The whole procedure for establishing the infection model is summarized in Figure 1. Initially two *C. elegans* strains were used as the hosts: the single *fem-3*(q96) mutant which is incapable of producing progeny at 25°C, and the *glp-4*(bn2); *sek-1*(km4) double mutant that is also unable to produce progeny at 25°C as well as being immunocompromised. In order to monitor the conidial ingestion and infection progression stages, we chose *A. fumigatus* Af293-dsRed strain with continuously red fluorescent signal (Jhingran et al., 2012), and the widely used parental strain KU80Δ for nematode model establishment (Table 1). Both Af293-dsRed and KU80Δ were able to kill and significantly reduce survival rate of *C. elegans fem-3*(q96) strain compared to heat-killed KU80Δ and *Escherichia coli* OP50, the preferred food of nematode (*P* < 0.0001) (Figure 2A, Supplementary Table 1). Interestingly, significant statistical difference was also observed between the survival rates of KU80Δ and Af293-dsRed (*P* < 0.0001) infections, indicating that Af293-dsRed is less virulent than KU80Δ. Similar susceptibility patterns and statistical values were obtained in the double mutant *glp-4*(bn2); *sek-1*(km4) host (Figure 2B, Supplementary Table 2). However, the worm survival rates with Af293-dsRed and KU80Δ in *glp-4*(bn2); *sek-1*(km4) mutant were much lower than that in *fem-3*(q96), demonstrating that the immunocompromised mutant worm is indeed more susceptible to fungal infections. To further substantiate the ability of our *C. elegans-A. fumigatus* infection model to be employed in evaluating pathogenicity of *A. fumigatus* strains, clinical strain Af293, which is also the parental strain of Af239-dsRed, was used for infection. The result showed that this strain displayed the same virulence as the Af293-dsRed strain, but was significantly less virulent than the KU80Δ strain (*P* < 0.0001) (Figure 2B, Supplementary Table 2). After 72 h of killing assay, the survival rates of *glp-4*(bn2); *sek-1*(km4) with Af293-dsRed, KU80Δ and Af293 were reduced to 6, 8, and 5%, respectively (Supplementary Table 2). The *glp-4*(bn2);*sek-1*(km4) strain was therefore used as the host strain for all the subsequent experiments.

**Table 1:** *A. fumigatus* strains used in this study.

**Strain**	**Parent or origin**	**Description**	**References/Sources**
Af293	Clinical strain	Lab “wild-type” strain	FGSC
Af293-dsRed	Af293	Red fluorescence in all growth stages Same radial growth on Sabouraud plates as Af293	Jhingran et al., 2012
KU80Δ	CEA17	High frequency of homologous recombination due to the deletion of *KU80* homolog	Da Silva Ferreira et al., 2006
Triple *ags*Δ	A1160	Triple deletion of α-1,3- glucan synthases genes Devoid α-(1,3)-glucan in the cell wall Restructured conidial cell wall Less pathogenic in murine aspergillosis model	Beauvais et al., 2013
Δ*pksP*	A1160	Lacking a functional polyketide synthase PksP, which is responsible for the initial step in DHN-melanin formation Producing white spores Attenuated virulence in murine aspergillosis model	Bayry et al., 2014; Kyrmizi et al., 2018
Δ*mrsA*	A1160	Activated reductive iron assimilation and siderophore-mediated iron acquisition Hypersusceptibility to azole and oxidative stresses Attenuated virulence in murine aspergillosis model	Long et al., 2016
Δ*leuB*	A1160	Growth defect that was cured by leucine or iron supplementation Activation of protease activity and autophagy Attenuated virulence in *Galleria mellonella*	Long et al., 2018
Δ*tptA*	A1160	Growth defects and decreased resistance to iron chelator Attenuated virulence in murine model	Huang et al., 2019
*Δafmid1*	A1160	Growth defects, delayed germination, and abnormal morphogenesis Sensitive to oxidative agents Enhanced virulence in murine aspergillosis model	Jiang et al., 2014

### Infection and Progression Stages of *A. fumigatus* in *C. elegans*

**Figure 3:**
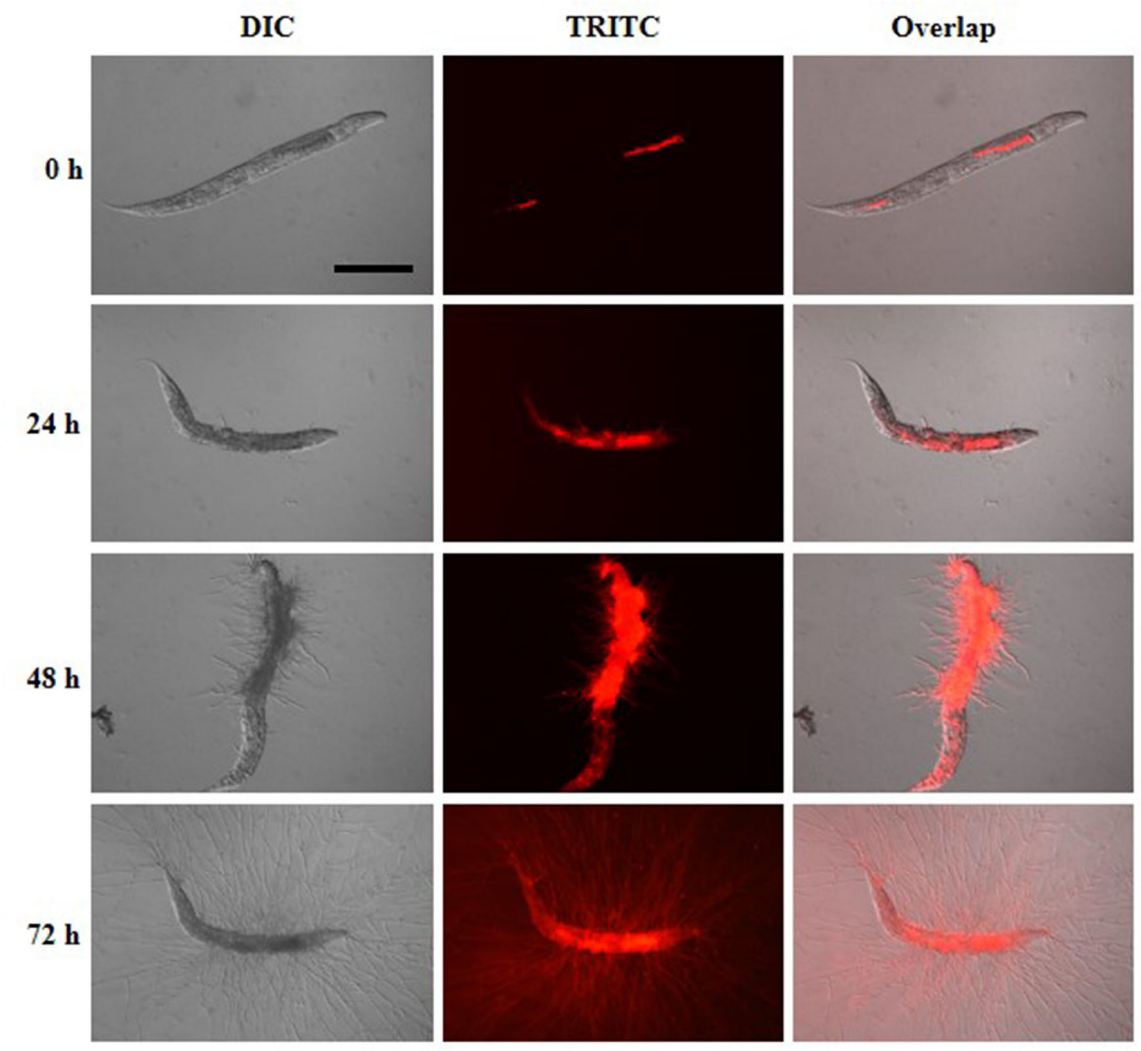
Infection and progression stages of Af293-dsRed in infection to *glp-4*(bn2); *sek-1*(km4) worms. Images were taken under DIC and TRITC channels at killing time of 0, 24, 48, and 72 h. Scale bar is 200 μm.

To understand the infection and progression stages of *A. fumigatus* in *C. elegans*, Af293-dsRed strain was used to infect *glp-4*(bn2); *sek-1*(km4) worms and spores progression was monitored using fluorescence microscope. As shown in Figure 3, the ingestion of conidia was clearly evident throughout the nematode intestine at the beginning of killing assay (Figure 3, Supplementary Figure 1). The conidia started to germinate with hyphae protruding from head, body and tail of *C. elegans* by 24 h (Figure 3, Supplementary Figure 2). When the killing assay reached 48 h, the hyphae became longer filaments protruding all over the worms' cuticle (Figure 3, Supplementary Figure 3). Furthermore, by 72 h, long hyphae diffused all through the body of the worms, making them appear as “ghosts” bodies (Figure 3). Survival of *C. elegans* was judged mainly by movement and sinusoidal shape as dead worms could not move and appeared straightened or slightly bent in shape, and mostly accompanied with fungal filaments.

### Pathogenicity Pattern of *A. fumigatus* Mutant Strains in *C. elegans* Model

**Figure 4:**
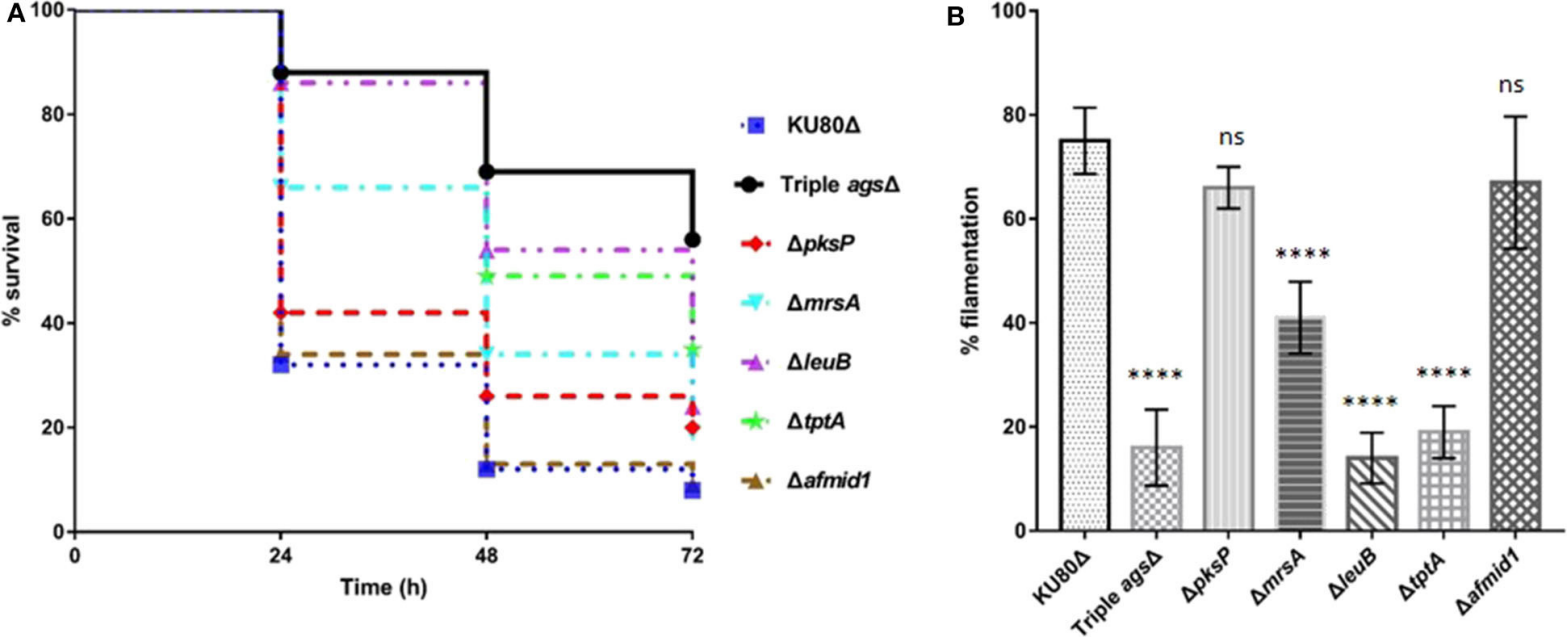
Survival curve and hyphal filamentation rate of *A. fumigatus* mutant strains in *C. elegans* model. **(A)**, Kaplan-Meier survival plots of *glp-4*(bn2); *sek-1*(km4) on solid BHI plates after 16 h pre-infection with conidia of indicate *A. fumigatus* mutant strains. **(B)**, Hyphal filamentation rates resulting from infection by indicated *A. fumigatus* mutant strains at 24 h in killing assay. Three biological repeats (each with triplicates) were conducted for each strain. One-way ANOVA was used for statistical analysis of filamentation rate, where *p* > 0.05 showing not significant, ns; *p* ≤ 0.0001 showing highly significant, ****, compared to the KU80Δ strain.

Following establishing the *C. elegans-A. fumigatus* infection model, we hypothesized that the model would be able to assess the pathogenicity of *A. fumigatus* mutant strains whose virulence have been tested in other infection models. Six mutant strains were collected including triple *ags*Δ mutant, Δ*pksP* mutant, Δ*mrsA* mutant, Δ*leuB* mutant, and Δ*tptA* mutant strains in which attenuated pathogenicity has been previously reported in mice or *G. mellonella* infection models (Pihet et al., 2009; Beauvais et al., 2013; Long et al., 2016, 2018; Huang et al., 2019), and Δ*afmid1* mutant with an augmented virulence reported in mice model (Jiang et al., 2014) (Table 1). The triple *ags*Δ mutant, obtained by sequential deletions of the three α-1,3 glucan synthase genes (*AGS1, AGS2*, and *AGS3*), is unable to synthesize α-(1, 3)-glucan, a major cell wall polysaccharide component (Henry et al., 2012; Beauvais et al., 2013). Compared to the parental KU80Δ strain, the triple *ags*Δ mutant displayed significant attenuated virulence (*P* < 0.0001) with the survival rate of 56 ± 6.9 by 72 h in killing assay (Figure 4A, Supplementary Table 3). Δ*pksP* is the knockout strain of polyketide synthase gene *pksp*, which is involved in cell wall melanin synthesis (Pihet et al., 2009; Bayry et al., 2014). There was statistically significant difference between the virulence of Δ*pksP* mutant and the KU80Δ (*P* < 0.05), with a higher virulence observed with KU80Δ indicating that this mutant is less virulent than KU80Δ. MrsA and TptA are mitochondrial transporters involved in iron adaptation and homeostasis whereas LeuB is a Zn2Cys6-type transcription factor for leucine biosynthesis and iron acquisition (Long et al., 2016, 2018; Huang et al., 2019). Reduced pathogenicity was recorded in our nematode model for Δ*mrsA*, Δ*leuB*, and Δ*tptA* strains, with high significant difference (*P* < 0.0001) when compared to KU80Δ (Figure 4A, Supplementary Table 3). However, Δ*afmid1* strain, which is devoid of the plasma ion channel for replenishing extracellular calcium (Jiang et al., 2014), showed no significant virulence difference with KU80Δ (*P* > 0.05) (Figure 4A, Supplementary Table 3). This observation is in contrast to its enhanced pathogenicity above KU80Δ reported in murine model (Jiang et al., 2014). Among the mutant strains, the triple *ags*Δ is the least virulent, followed by Δ*tptA* and Δ*leuB* strains, and then the less attenuated Δ*pksP* and Δ*mrsA* strains. Interestingly, the hyphal filamentation rates at 24 h of killing assay followed the same pattern as the survival rates. The Δ*afmid1* mutant and the parental strain KU80Δ had the highest filamentation rate whereas the triple *ags*Δ mutant had the least (Figure 4B, Supplementary Table 3). It was observed that the more virulent the strain is, the higher the hyphal filamentation rate, suggesting that the death of the infected worms was mainly due to the germination and hyphal growth of conidia inside the worms which eventually led to puncture of worms when the filaments started to protrude. We noted that the filamentation of these mutant strains in *C. elegans* model did not correlate with *in vitro* growth in BHI medium, of which the triple *ags*Δ mutant exhibited the fastest germination, followed by KU80Δ, Δ*pksp*, Δ*leuB*, and Δ*afmid1* mutant strains, while the Δ*mrsA* and Δ*tptA* mutant strains were the slowest (Supplementary Figure 4).

### Evaluation of Antifungal Drug Efficacy in *C. elegans-A. fumigatus* Infection Model

**Figure 5:**
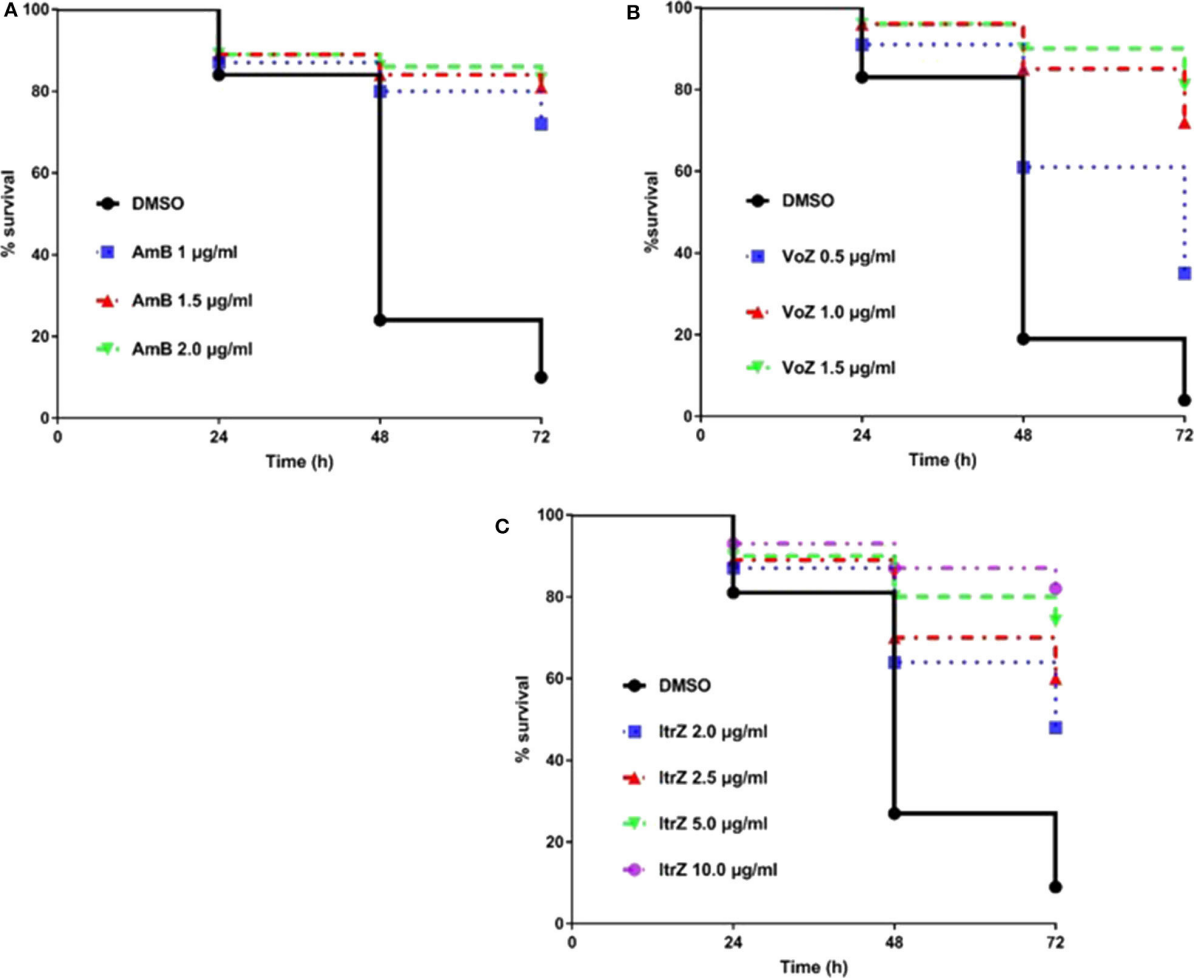
Kaplan-Meier survival plots of *C. elegans* infected by KU80Δ in the presence of antifungal drugs. The *glp-4*(bn2); *sek-1*(km4) worms were pre-infected with KU80Δ for 8 h then transferred into liquid killing media containing different concentrations of AmB **(A)**, VoZ **(B)**, and ItrZ **(C)**. Three biological repeats (each with triplicates) were conducted for each concentration.

Having established and confirmed the virulence pattern of our *C. elegans*-*A. fumigatus* infection model, the next challenge was to determine if clinically used antifungal drugs could rescue the aspergillosis infection and death in the nematode model. To achieve a uniformity exposure of the worms to the antifungals as well as ensure sufficient bioavailability of the drug, we optimized the pre-infection time for the liquid killing assay. Pre-infections of 8, 12, and 16 h on solid medium were tested and 8 h was chosen as the best duration for the worms to ingest sufficient conidia while also allowing adequate antifungal treatment test. Amphotericin B (AmB), itraconazole (ItrZ), and voriconazole (VoZ) were used in our *C. elegans* antifungal assay since they are the three commonly used drugs for aspergillosis treatment in clinical settings. All the three drugs showed excellent efficacy and increased the survival of infected worms when compared to the DMSO control treatments. AmB exhibited statistically significant rescue at concentration of 1 μg/ml or higher when compared to DMSO control (*P* < 0.0001) (Figure 5A, Supplementary Table 4). Similar significant rescue was obtained for ItrZ and VoZ at ≥2 μg/ml and ≥0.5 μg/ml, respectively, compared to DMSO control (*P* < 0.0001) (Figures 5B,C, Supplementary Tables 5, 6). The rescue effects of these antifungal drugs illustrated that our *C. elegans-A. fumigatus* infection model is suitable for evaluating antifungal drug efficacy.

### Killing Mode of Antifungal Drugs in *C. elegans-A. fumigatus* Infection Model

**Figure 6:**
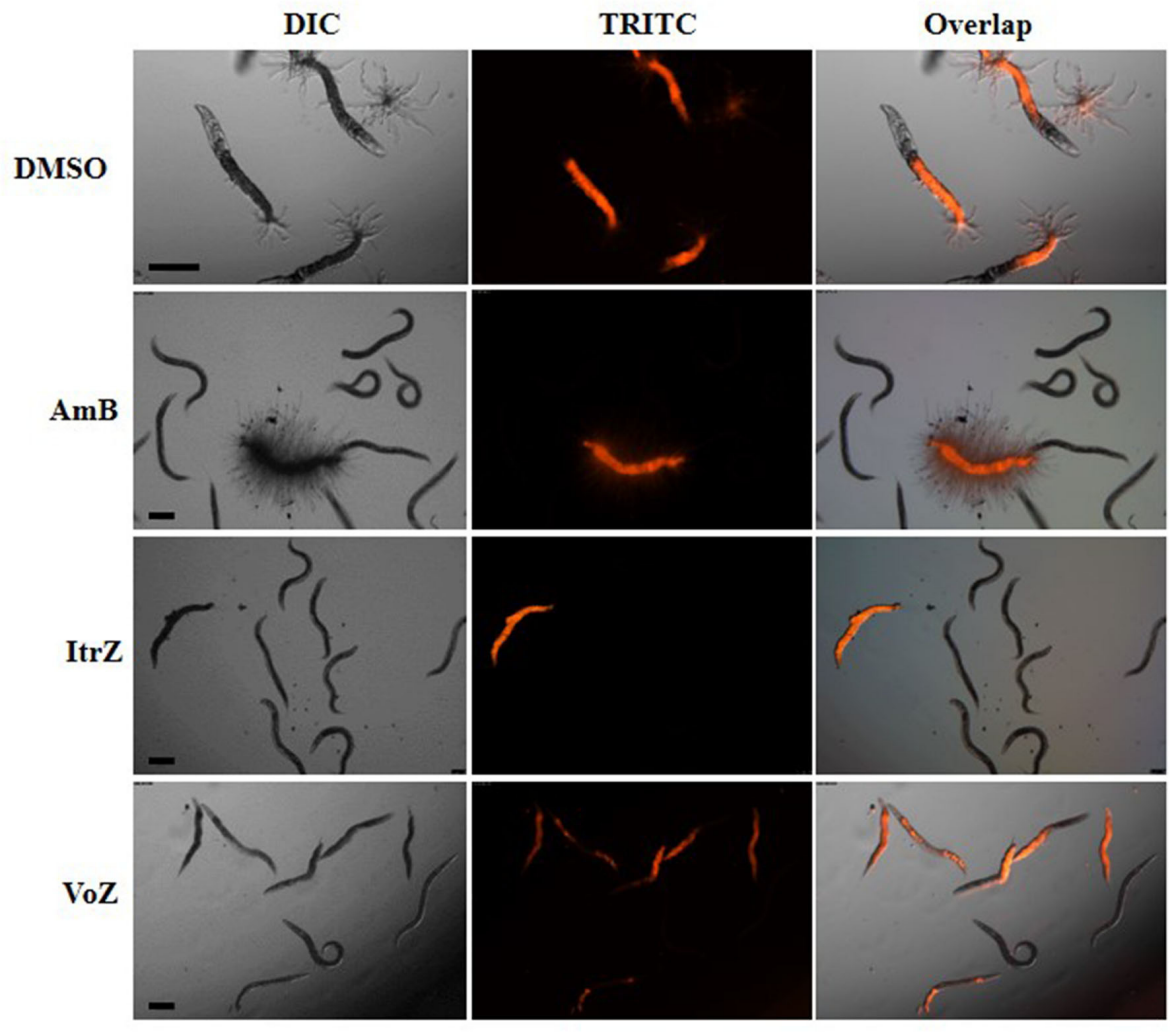
Effect of antifungal treatment on Af293-dsRed infection to *glp-4*(bn2); *sek-1*(km4) worms. Images were taken under DIC and TRITC channels from DMSO treatment by 24 h, 1.5 μg/ml AmB treatment by 48 h, 2 μg/ml ItrZ treatment by 72 h and 0.5 μg/ml VoZ treatment by 72 h. Scale bar is 200 μm.

To analyze the killing mode of the antifungal drugs in *C. elegans-A. fumigatus* infection model, Af293-dsRed strain was used for infection of *glp-4*(bn2);*sek-1*(km4) worms. As shown in Figure 6, worms treated with DMSO had hyphal filaments protruding from their cuticles even at 24 h in killing assay. The fungal load from worms treated with the three drugs was significantly reduced as shown from the worm numbers with fluorescent signals (Figure 6). Moreover, dead worms from the AmB treated group possessed advanced hyphal growth whereas dead worms from ItrZ or VoZ treated group had strong accumulated fluorescent signals inside the body but without protruding hyphal filaments (Figure 6). It is interesting to note that ItrZ and VoZ exhibited superior antifungal activity against *A. fumigatus* by preventing the hyphal growth in *C. elegans* model when compared to AmB treatment.

## Discussion

*C. elegans* as a model organism has been applied for evaluating pathogenicity, studying host-pathogen interactions, testing the efficacy of potential anti-infective compounds, and screening new antimicrobial agents against pathogenic bacteria and fungi. Okoli and Bignell had initially reported that *C. elegans-*based infection model could be applied to the filamentous fungus *A. fumigatus* (Okoli and Bignell, 2015). However, sufficient details on the infection process has not been provided to validate the model.

In this study through a series of optimization trials we developed a stable and efficient *C. elegans-A. fumigatus* infectious model. At the beginning of setting up the infection of the worms with *A. fumigatus* spores, fluorescence imaging showed that each of the single and double mutant worms all ingested varying amount of conidia. In addition to this challenge, it was also laborious and difficult to count the exact spore numbers from 100 or 1,000 of worms, which would involve crushing the worms and separating spores from worm lysate for counting. However, we noticed that the conidia concentration, worm numbers and the pre-infection time affected the amount of ingested fungal spores and survival of worms. Therefore, we standardized the concentration of spores and worm numbers, fixed the pre-infection time as 16 h for pathogenicity evaluation and applied conidia in four different points on the pre-infection plates to enhance the chances of conidia ingestion by the worms. As shown in Supplementary Tables 1–6, the survival rate of worms in killing assay displayed acceptable standard deviations from three biological repeats of each with triplicates, confirming the repeatability of the infection assay using our optimized protocol even if each worm may not ingest the same amount of spores. A device of filter membrane-attached-on-tube was developed to allow fast extensive worms-spores separation washing, thereby enabling better removal of conidia that were not ingested by the worms. The extensive washing after pre-infection also assisted easier observation of the killing assay without much obstruction from *in vitro* aggressive hyphal growth (Figure 1). Fluorescent Af293-dsRed strain provided further support to our findings that infection could emanate from any part of the worms including head, neck, abdomen, and tail regions through hyphal filaments protrusion (Figure 3), not only through the tail region as initially reported (Okoli and Bignell, 2015).

Previous studies have demonstrated that hyphal filamentation is an important virulence factor in fungal pathogenicity in mammal (Mitchell et al., 2007; Ariyachet et al., 2013; Ghosh et al., 2015) and nematode models (Breger et al., 2007; Pukkila-Worley et al., 2009; Huang et al., 2014). Similarly, in our *C. elegans-A. fumigatus* infection model, the *in vivo* germination and growth of conidia into filaments led to the quick death of worms (as the epidermal cuticles were disrupted) as well as limited worm's movement due to worm-hyphae adhesion to the medium surface. In both the single and double mutant worms, strain KU80Δ exhibited higher virulence than Af293 and Af293-dsRed strain, corresponding to higher hyphal filamentation rate at 24 h in killing assay (Figure 2, Supplementary Tables 1, 2). To further corroborate the role of hyphal filamentation in the virulence of *A. fumigatus* in *C. elegans*, our results from heat-killed conidia gave no hyphal filamentation as expected and displayed no difference from *E.coli* OP50 with regard to the survival rate of worms (Figure 2, Supplementary Tables 1, 2).

Assessing the pathogenicity of *A. fumigatus* strains is one of the main objective of setting up the nematode model. Among the selected six *A. fumigatus* mutant strains, triple *ags*Δ mutant, Δ*pksP*, Δ*mrsA*, and Δ*tptA* mutants all displayed significant attenuated virulence compared to the parental KU80Δ strain (Figure 4), consistent with their virulence patterns already shown in immunocompromised murine model of aspergillosis (Beauvais et al., 2013; Bayry et al., 2014; Long et al., 2016; Huang et al., 2019). Similar attenuated virulence was obtained for Δ*leuB* mutant in our *C. elegans* model which is in agreement with previously described pathogenicity pattern in *G. mellonella* model (Long et al., 2018). An exception was the Δ*afmidl* mutant which was hypervirulent in murine model (Jiang et al., 2014) but exhibited no significant difference in our *C. elegans* model when compared to the KU80Δ strain. Currently we could not explain why Δ*afmidl* virulence was not enhanced in *C. elegans* model but we strongly suspect that it could be due to the vast differences in *in vivo* environment between the nematode and mice, including the immune system and concentration of calcium ion, which requires further investigation. Again the correlation between virulence and hyphal filamentation was confirmed in infections caused by the six mutant strains (Figure 4B, Supplementary Table 3). However, it is noteworthy that some *A. fumigatus* strains, such as the Δ*leuB* and Δ*tptA* mutant strains, did not produce much filamentation at 24 h but eventually became relatively virulent by 72 h in killing assay, implying that hyphal filamentation is not the sole factor responsible for virulence, which further buttresses the need for molecular characterization. To the best of our knowledge, this is the first time that the pathogenicity of *A. fumigatus* strains in *C. elegans* infection model is being compared with that from other animal models. Our stable and reliable nematode infection model would be suitable for evaluating the virulence of more *A. fumigatus* mutant strains, such as mutants from the ongoing COFUN project, which plans to generate knockout mutants of all the coding genes in *A. fumigatus* (https://www.phe-culturecollections.org.uk/products/fungi/cofun-aspergillus-fumigatus-gene-wide-knock-out-collection.aspx).

The major drawbacks of the nematode model system include the inability to culture at 37°C and the absence of an adaptive immune response. Even the innate immune responses against pathogenic organisms in murine and *C. elegans* are different. Unlike in mammalian systems in which the transcription factor NF-κB, the Toll-like receptor (TLR) adaptor protein MYD88 and other components of the TLR signaling pathway are key to the innate immune system, *C. elegans* lacks these components. The *C. elegans* immune system however, mounts several conserved signaling pathways, including the p38 mitogen activated protein kinase (MAPK) cassette, the transforming growth factor (TGFβ), the insulin-like DAF-2-DAF-16, as well as the bZIP transcription factor ZIP-2 to defend against pathogens (Pukkila-Worley and Ausubel, 2012). Nevertheless, it is still unclear what the exact pathogenic triggers are and how the *C. elegans* detected and responded to pathogens. In murine model, the conidial cell wall of the triple *ags*Δ mutant was covered by a glycoprotein matrix, and PAMPs such as chitin and β-glucan were exposed, thus stimulating host immune response, which led to reduced virulence (Beauvais et al., 2013). Attenuated virulence of the Δ*pksp* mutant in murine aspergillosis model is reported to be due to β-glucan exposure and the activated autophagy pathway of the LC3-associated phagocytosis (LAP) (Akoumianaki et al., 2016). The immune responses caused by the other four mutant strains are not clear yet in murine or *G. mellonella* models. Nevertheless, it will be desirable to uncover the immune defense of *C. elegans* in more detail by combining histological dissection of intestine epithelium cell structure and global transcription analysis of worms infected by KU80Δ and those mutant strains, particularly by the triple *ags*Δ mutant and the Δ*pksp* mutant.

*C. elegans* model has been utilized in the evaluation of the efficacy of antifungal agents against infections caused by dimorphic fungi, such as *C. albicans, C. krusei, C. parapsilosis*, and *P. marneffei* (Breger et al., 2007; Huang et al., 2014) but not with the filamentous fungus, *A. fumigatus*. We have also demonstrated the possibility of adopting the *C. elegans* model for *in vivo* evaluation of antifungal agents on nematode aspergillosis. Shorter pre-infection time and liquid killing medium were applied in the assay to assess drug effectiveness. The minimum treatment doses of AmB, ItrZ, and VoZ in *C. elegans* were 1, 2, and 0.5 μg/ml, respectively, close to the *in vitro* MIC values of the tested antifungal drugs (Subcommittee on Antifungal Susceptibility Testing of the ESCMID European Committee for Antimicrobial Susceptibility Testing, 2008). The evaluation assay can easily be scaled up from 24-well-plates to 96-well or 384-well-plates for high-throughput screening of other bioactive compounds against *A. fumigatus*. Interestingly the killing mode of antifungal drugs in *C. elegans* model was different from that in the *in vitro* culture. The macrolide polyene AmB is a well-known broad-spectrum fungicidal agent (Hamill, 2013; Ashu et al., 2018) while azole drugs, i.e., VoZ and ItrZ, are mainly fungistatic against *A. fumigatus* (Meletiadis et al., 2007). However, visible hyphal filamentation from a number of infected worms graduated to “ghost stage” under AmB treatment whereas only diffused internal hyphal growth and early germinated conidia were observed from worms treated with VoZ and ItrZ (Figure 6). This is suggestive that VoZ and ItrZ may be an attractive alternative to AmB in treatment of invasive pulmonary aspergillosis and reducing occurrence of disseminated aspergillosis in *in vivo* applications.

In summary, our established *C. elegans*-*A. fumigatus* infection model could serve as a preliminary screening for pathogenic phenotypes of *A. fumigates* strains. The application of this model to evaluate the efficacy of antifungal agents on nematode aspergillosis could be the groundwork to develop future antifungal agents against *A. fumigatus*.

## Materials and Methods

### Strains, Media, and Culture Conditions

The *A. fumigatus* strains used in this study are summarized in Table 1. The strains were grown on *A. fumigatus* complete medium (Subcommittee on Antifungal Susceptibility Testing of the ESCMID European Committee for Antimicrobial Susceptibility Testing, 2008) slants composed of 1 g/l yeast extract, 2 g/l peptone, 10 g/l glucose, 1.5 g/l casein hydrolysate acid, 1 ml/l trace element solution, 20 ml/l 50X salt solution. After incubation at 37°C for 24–72 h (depending on mutant strains) conidia were harvested with 0.2% (v/v) Tween 20 aqueous solution, centrifuged and then Tween 20 aqueous solution was decanted out. The conidia were then re-suspended in M9 buffer and standardized to 1.25 × 10^8^ spores/ml in Eppendorf tube.

The *C. elegans* strains *fem-3*(q96) and *glp-4*(bn2); *sek-1*(km4) were used in this study. The *fem-3*(q96) and *glp-4(bn2)* mutations are temperature sensitive and thus prevents them from producing progeny at 25°C while the *sek-1*(*km4*) mutation makes the worms immunocompromised. Worms were grown at 15°C in 90 mm Nematode Growth Medium (Fedorova et al., 2008) plates seeded with *E. coli* OP50.

### Setting up *C. elegans-A. fumigatus* Infection Model

NGM medium plus antibiotics (NGM+) was used for pre-infection assay according to methods modified from Okoli and Bignell (2015). Ampicillin, streptomycin, and kanamycin were added to NGM at 100, 100, and 45 μg/ml, respectively. Unstarved worms for at least three generations were filtered through 11 μm pore-sized membrane filter (Merck Millipore Ltd.) using M9, to collect L1 worms for synchronization. Filtrated L1 worms were spread on NGM plates seeded with OP50, and then incubated at 25°C for 48 h for synchronization.

Synchronized L4 worms were washed off from the NGM plates with M9 and transferred to 50 ml tube to sediment for 10 min. The M9-OP50 mix was gently removed using micropipette. Three more sedimentation washes were performed and then standardized worms (about 200–500) were dispensed into triplicate 90 mm NGM+ plates and gently spread to dry. Worms were allowed to move around on the plates for 30 min before 25 μl of standardized conidia (1.25 × 10^8^ spores/ml) were added at the four cardinal points of each NGM+ plates making a total of 100 μl conidia per plate. The plates were then incubated at 25°C for 16 h pre-infection period to allow worms ingest conidia. Control was setup with OP50 instead of conidia.

A 30% Brain Heart Infusion (BHI) plus 200 μg/ml ampicillin, 200 μg/ml streptomycin, and 90 μg/ml kanamycin in M9 solid medium (referred to as BHI+S) was used for solid killing assay. Pre-infected worms were washed off with M9 into 50 ml tube. Then we developed a hand-made “filter membrane-attached-on-tube” device with a 35 μm pore-sized membrane (Sango Biotech) designed into 15 ml Eppendorf tubes to wash out conidia that were not ingested by worms. The filter separated conidia that were not ingested from *C. elegans* to drastically reduce the amount of conidia that would otherwise interfere with the experiment when germinated on BHI+S plates. About 80–200 washed worms were dispensed into 90 mm BHI+S plates in triplicates. Living and dead worms with or without hyphal filament were recorded at 24 h in killing assay using dissecting microscope (Motic SMZ-168 series) whereas only dead and living worm numbers were recorded at 48 and 72 h of killing assay.

### Microscopic Imaging

We used two approaches to prepare our worms for fluorescent microscopy. The first approach majorly applied to worms at 0 h in killing assay (immediately after 16 h of pre-infection). We made 2% agarose pad on slides and used worm picker to randomly collect worms from BHI+S plates. Worms were transferred to the agarose gel in M9 plus levamisole, then observed and recorded under both fluorescence and DIC channels of Leica upright microscope (Leica Microsystems, STP 8000). The second approach involved cutting out and transferring the BHI+S agar with worms to slides between 24 and 72 h in killing assay. This was very necessary as protrusion of hyphal filaments made worms attached to the medium.

### The Procedure for Evaluating Antifungal Drugs Efficacy

The 8 h Pre-infection time and liquid killing assay were chosen for antifungal drugs evaluation and BHI+S without agar (referred to as BHI+L) was used as the killing medium. Amphotericin B, itraconazole, and voriconazole (MedChemExpress) were prepared in DMSO and diluted to 20 μg/ml with BHI+L (referred to as BHI+L+). Antifungal drug concentrations ranging from 0.5 to 2.0 μg/ml for amphotericin B, 1.0–2.0 μg/ml for voriconazole, and 1.0–10 μg/ml for itraconazole were utilized in 24-well-plates. BHI+L+ were dispensed into triplicates of 24-well-plates to a volume of 320 μl. Then 80 μl of pre-infected worms (~30–60) were added into each well to make the final volume of 400 μl. DMSO was used as control. Dead and living worm numbers were recorded using Leica inverted microscope (Leica DMC 5400). Antifungal phenotypic images were captured by the inverted microscope under both fluorescent and DIC channels.

### Data Analysis

All infection experiments were performed in triplicates and all the experimental numerical data were expressed as the means ± S.D. The Kaplan-Meier survival curves were plotted by GraphPad Prism 7.0. Statistical *P-*values for survival rates were calculated by Log-rank (Mantel-Cox) test. One-way ANOVA was used for statistical analysis of filamentation rates. Images analysis were performed using ImageJ software.

## Data Availability Statement

The raw data supporting the conclusions of this article will be made available by the authors, without undue reservation, to any qualified researcher.

## Author Contributions

WF and BW conceptualized the study. WF helped with data curation. CA and QQ did the formal analysis. CA, QQ, AM, and JO carried out the investigation. QQ and WF contributed to software. CJ, WF, and BW supervised the study. CA wrote the original draft. AO, WF, and BW reviewed and edited the manuscript. All authors contributed to the article and approved the submitted version.

## Conflict of Interest

The authors declare that the research was conducted in the absence of any commercial or financial relationships that could be construed as a potential conflict of interest.

## References

[B1] Abdolrasouli A. Scourfield A. Rhodes J. Shah A. Elborn J. S. Fisher M. C.. (2018). High prevalence of triazole resistance in clinical *Aspergillus fumigatus* isolates in a specialist cardiothoracic centre. Int. J. Antimicrob. Agents 52, 637–642. 10.1016/j.ijantimicag.2018.08.004 30103005

[B2] Akoumianaki T. Kyrmizi I. Valsecchi I. Gresnigt M. S. Samonis G. Drakos E.. (2016). Aspergillus cell wall melanin blocks LC3-associated phagocytosis to promote pathogenicity. Cell Host Microbe 19, 79–90. 10.1016/j.chom.2015.12.002 26749442

[B3] Ariyachet C. Solis N. V. Liu Y. Prasadarao N. V. Filler S. G. Mcbride A. E. (2013). SR-like RNA-binding protein Slr1 affects Candida albicans filamentation and virulence. Infect. Immun. 81, 1267–1276. 10.1128/IAI.00864-12 23381995 PMC3639594

[B4] Ashu E. E. Korfanty G. A. Samarasinghe H. Pum N. You M. Yamamura D.. (2018). Widespread amphotericin B-resistant strains of *Aspergillus fumigatus* in Hamilton, Canada. Infect. Drug Resist. 11, 1549–1555. 10.2147/IDR.S170952 30288065 PMC6160276

[B5] Bayry J. Beaussart A. Dufrene Y. F. Sharma M. Bansal K. Kniemeyer O.. (2014). Surface structure characterization of *Aspergillus fumigatus* conidia mutated in the melanin synthesis pathway and their human cellular immune response. Infect. Immun. 82, 3141–3153. 10.1128/IAI.01726-14 24818666 PMC4136205

[B6] Beauvais A. Bozza S. Kniemeyer O. Formosa C. Balloy V. Henry C.. (2013). Deletion of the alpha-(1,3)-glucan synthase genes induces a restructuring of the conidial cell wall responsible for the avirulence of *Aspergillus fumigatus*. PLoS Pathog. 9:e1003716. 10.1371/annotation/05c0ca66-4ed9-4c04-96c6-3addac835e04 24244155 PMC3828178

[B7] Beer K. D. Farnon E. C. Jain S. Jamerson C. Lineberger S. Miller J.. (2018). Multidrug-resistant *Aspergillus fumigatus* carrying mutations linked to environmental fungicide exposure - three States, 2010-2017. MMWR Morb. Mortal. Wkly. Rep. 67, 1064–1067. 10.15585/mmwr.mm6738a5 30260939 PMC6188124

[B8] Breger J. Fuchs B. B. Aperis G. Moy T. I. Ausubel F. M. Mylonakis E. (2007). Antifungal chemical compounds identified using a C. elegans pathogenicity assay. PLoS Pathog. 3:e18. 10.1371/journal.ppat.0030018 17274686 PMC1790726

[B9] Da Silva Ferreira M. E. Kress M. R. Savoldi M. Goldman M. H. Hartl A. Heinekamp T.. (2006). The akuB(KU80) mutant deficient for nonhomologous end joining is a powerful tool for analyzing pathogenicity in *Aspergillus fumigatus*. Eukaryotic Cell 5, 207–211. 10.1128/EC.5.1.207-211.2006 16400184 PMC1360264

[B10] Darby C. Cosma C. L. Thomas J. H. Manoil C. (1999). Lethal paralysis of caenorhabditis elegans by *Pseudomonas aeruginosa*. Proc. Natl. Acad. Sci. U.S.A. 96, 15202–15207. 10.1073/pnas.96.26.15202 10611362 PMC24797

[B11] Darling B. A. Milder E. A. (2018). Invasive aspergillosis. Pediatr. Rev. 39, 476–478. 10.1542/pir.2017-0129 30171061

[B12] Fang W. Latge J. P. (2018). Microbe profile: *Aspergillus fumigatus*: a saprotrophic and opportunistic fungal pathogen. Microbiology 164, 1009–1011. 10.1099/mic.0.000651 30066670 PMC6152418

[B13] Fedorova N. D. Khaldi N. Joardar V. S. Maiti R. Amedeo P. Anderson M. J.. (2008). Genomic islands in the pathogenic filamentous fungus *Aspergillus fumigatus*. PLoS Genet. 4:e1000046. 10.1371/journal.pgen.1000046 18404212 PMC2289846

[B14] Geissel B. Loiko V. Klugherz I. Zhu Z. Wagener N. Kurzai O.. (2018). Azole-induced cell wall carbohydrate patches kill *Aspergillus fumigatus*. Nat. Commun. 9:3098. 10.1038/s41467-018-05497-7 30082817 PMC6078979

[B15] Ghosh A. K. Wangsanut T. Fonzi W. A. Rolfes R. J. (2015). The GRF10 homeobox gene regulates filamentous growth in the human fungal pathogen Candida albicans. FEMS Yeast Res. 15:fov093. 10.1093/femsyr/fov093 26472755 PMC4705307

[B16] Gomez-Lopez A. Forastiero A. Cendejas-Bueno E. Gregson L. Mellado E. Howard S. J.. (2014). An invertebrate model to evaluate virulence in *Aspergillus fumigatus*: the role of azole resistance. Med. Mycol. 52, 311–319. 10.1093/mmy/myt022 24577012

[B17] Hagiwara D. Arai T. Takahashi H. Kusuya Y. Watanabe A. Kamei K. (2018). Non-cyp51A azole-resistant *Aspergillus fumigatus* Isolates with mutation in HMG-CoA reductase. Emerging Infect. Dis. 24, 1889–1897. 10.3201/eid2410.180730 30226177 PMC6154143

[B18] Hagiwara D. Miura D. Shimizu K. Paul S. Ohba A. Gonoi T.. (2017). A Novel Zn2-Cys6 transcription factor AtrR plays a key role in an azole resistance mechanism of *Aspergillus fumigatus* by co-regulating cyp51A and cdr1B expressions. PLoS Pathog. 13:e1006096. 10.1371/journal.ppat.1006096 28052140 PMC5215518

[B19] Hamill R. J. (2013). Amphotericin B formulations: a comparative review of efficacy and toxicity. Drugs 73, 919–934. 10.1007/s40265-013-0069-4 23729001

[B20] Henry C. Latge J. P. Beauvais A. (2012). alpha1,3 glucans are dispensable in *Aspergillus fumigatus*. Eukaryotic Cell 11, 26–29. 10.1128/EC.05270-11 22058140 PMC3255942

[B21] Huang J. Ma Z. Zhong G. Sheppard D. C. Lu L. Zhang S. (2019). The mitochondrial thiamine pyrophosphate transporter TptA promotes adaptation to low iron conditions and virulence in fungal pathogen *Aspergillus fumigatus*. Virulence 10, 234–247. 10.1080/21505594.2019.1596505 30880633 PMC6527022

[B22] Huang X. Li D. Xi L. Mylonakis E. (2014). Caenorhabditis elegans: a simple nematode infection model for Penicillium marneffei. PLoS ONE 9:e108764. 10.1371/journal.pone.0108764 25268236 PMC4182626

[B23] Jansen W. T. Bolm M. Balling R. Chhatwal G. S. Schnabel R. (2002). Hydrogen peroxide-mediated killing of Caenorhabditis elegans by Streptococcus pyogenes. Infect. Immun. 70, 5202–5207. 10.1128/IAI.70.9.5202-5207.2002 12183571 PMC128270

[B24] Jhingran A Mar K. B. Kumasaka D. K. Knoblaugh S. E. Ngo L. Y. Segal B. H.. (2012). Tracing conidial fate and measuring host cell antifungal activity using a reporter of microbial viability in the lung. Cell Rep. 2, 1762–1773. 10.1016/j.celrep.2012.10.026 23200858 PMC3712646

[B25] Jiang H. Shen Y. Liu W. Lu L. (2014). Deletion of the putative stretch-activated ion channel Mid1 is hypervirulent in *Aspergillus fumigatus*. Fungal Genet. Biol. 62, 62–70. 10.1016/j.fgb.2013.11.003 24239700

[B26] Johnson C. H. Ayyadevara S. Mcewen J. E. Shmookler Reis R. J. (2009). Histoplasma capsulatum and Caenorhabditis elegans: a simple nematode model for an innate immune response to fungal infection. Med. Mycol. 47, 808–813. 10.3109/13693780802660532 20028234

[B27] Kong C. Yehye W. A. Abd Rahman N. Tan M. W. Nathan S. (2014). Discovery of potential anti-infectives against *Staphylococcus aureus* using a Caenorhabditis elegans infection model. BMC Complement. Altern. Med. 14:4. 10.1186/1472-6882-14-4 24393217 PMC3893568

[B28] Kyrmizi I. Ferreira H. Carvalho A. Figueroa J. A. L. Zarmpas P. Cunha C.. (2018). Calcium sequestration by fungal melanin inhibits calcium-calmodulin signalling to prevent LC3-associated phagocytosis. Nat Microbiol. 3, 791–803. 10.1038/s41564-018-0167-x 29849062

[B29] Labrousse A. Chauvet S. Couillault C. Kurz C. L. Ewbank J. J. (2000). Caenorhabditis elegans is a model host for Salmonella typhimurium. Curr. Biol. 10, 1543–1545. 10.1016/S0960-9822(00)00833-2 11114526

[B30] Lionakis M. S. Kontoyiannis D. P. (2012). Drosophila melanogaster as a model organism for invasive aspergillosis. Methods Mol. Biol. 845, 455–468. 10.1007/978-1-61779-539-8_32 22328395 PMC4045217

[B31] Long N. Orasch T. Zhang S. Gao L. Xu X. Hortschansky P.. (2018). The Zn2Cys6-type transcription factor LeuB cross-links regulation of leucine biosynthesis and iron acquisition in *Aspergillus fumigatus*. PLoS Genet. 14:e1007762. 10.1371/journal.pgen.1007762 30365497 PMC6221358

[B32] Long N. Xu X. Qian H. Zhang S. Lu L. (2016). A putative mitochondrial iron transporter MrsA in *Aspergillus fumigatus* plays important roles in azole-, oxidative stress responses and virulence. Front. Microbiol. 7:716. 10.3389/fmicb.2016.00716 27433157 PMC4922219

[B33] Meletiadis J. Antachopoulos C. Stergiopoulou T. Pournaras S. Roilides E. Walsh T. J. (2007). Differential fungicidal activities of amphotericin B and voriconazole against Aspergillus species determined by microbroth methodology. Antimicrob. Agents Chemother. 51, 3329–3337. 10.1128/AAC.00345-07 17576838 PMC2043246

[B34] Mitchell B. M. Wu T. G. Jackson B. E. Wilhelmus K. R. (2007). Candida albicans strain-dependent virulence and Rim13p-mediated filamentation in experimental keratomycosis. Invest. Ophthalmol. Vis. Sci. 48, 774–780. 10.1167/iovs.06-0793 17251477

[B35] Mylonakis E. Ausubel F. M. Perfect J. R. Heitman J. Calderwood S. B. (2002). Nonlinear partial differential equations and applications: Killing of Caenorhabditis elegans by Cryptococcus neoformans as a model of yeast pathogenesis. Proc. Natl. Acad. Sci. U.S.A. 99, 15675–15680. 10.1073/pnas.232568599 12438649 PMC137775

[B36] Nakamura I. Kanasaki R. Yoshikawa K. Furukawa S. Fujie A. Hamamoto H.. (2017). Discovery of a new antifungal agent ASP2397 using a silkworm model of *Aspergillus fumigatus* infection. J. Antibiot. 70, 41–44. 10.1038/ja.2016.106 27577982

[B37] Okoli I. Bignell E. (2015). Caenorhabditis elegans-*Aspergillus fumigatus* (nematode-mould) model for study of fungal pathogenesis. Br. Microbiol. Res. J. 7, 93–99. 10.9734/BMRJ/2015/15838

[B38] Okoli I. Coleman J. J. Tampakakis E. An W. F. Holson E. Wagner F.. (2009). Identification of antifungal compounds active against Candida albicans using an improved high-throughput Caenorhabditis elegans assay. PLoS ONE 4:e7025. 10.1371/journal.pone.0007025 19750012 PMC2737148

[B39] Paulussen C. Boulet G. Bosschaerts T. Cos P. Fortin A. Maes L. (2015). Efficacy of oleylphosphocholine (OlPC) *in vitro* and in a mouse model of invasive aspergillosis. Mycoses 58, 127–132. 10.1111/myc.12286 25590577

[B40] Pihet M. Vandeputte P. Tronchin G. Renier G. Saulnier P. Georgeault S.. (2009). Melanin is an essential component for the integrity of the cell wall of *Aspergillus fumigatus* conidia. BMC Microbiol. 9:177. 10.1186/1471-2180-9-177 19703288 PMC2740851

[B41] Prigitano A. Esposto M. C. Biffi A. De Lorenzis G. Favuzzi V. Koncan R.. (2017). Triazole resistance in *Aspergillus fumigatus* isolates from patients with cystic fibrosis in Italy. J. Cyst. Fibros. 16, 64–69. 10.1016/j.jcf.2016.06.006 27356848

[B42] Prigitano A. Esposto M. C. Romano L. Auxilia F. Tortorano A. M. (2019). Azole-resistant *Aspergillus fumigatus* in the Italian environment. J. Glob. Antimicrob. Resist. 16, 220–224. 10.1016/j.jgar.2018.10.017 30367993

[B43] Pukkila-Worley R. Ausubel F. M. (2012). Immune defense mechanisms in the Caenorhabditis elegans intestinal epithelium. Curr. Opin. Immunol. 24, 3–9. 10.1016/j.coi.2011.10.004 22236697 PMC3660727

[B44] Pukkila-Worley R. Peleg A. Y. Tampakakis E. Mylonakis E. (2009). Candida albicans hyphal formation and virulence assessed using a Caenorhabditis elegans infection model. Eukaryotic Cell 8, 1750–1758. 10.1128/EC.00163-09 19666778 PMC2772404

[B45] Sharma C. Nelson-Sathi S. Singh A. Radhakrishna Pillai M. Chowdhary A. (2019). Genomic perspective of triazole resistance in clinical and environmental *Aspergillus fumigatus* isolates without cyp51A mutations. Fungal Genet. Biol. 132:103265. 10.1016/j.fgb.2019.103265 31465846

[B46] Stevens D. A. Melikian G. L. (2011). Aspergillosis in the ‘nonimmunocompromised’ host. Immunol. Invest. 40, 751–766. 10.3109/08820139.2011.614307 21985304

[B47] Styer K. L. Hopkins G. W. Bartra S. S. Plano G. V. Frothingham R. Aballay A. (2005). Yersinia pestis kills Caenorhabditis elegans by a biofilm-independent process that involves novel virulence factors. EMBO Rep. 6, 992–997. 10.1038/sj.embor.7400516 16170309 PMC1369189

[B48] Subcommittee on Antifungal Susceptibility Testing of the ESCMID European Committee for Antimicrobial Susceptibility Testing (2008). EUCAST technical note on the method for the determination of broth dilution minimum inhibitory concentrations of antifungal agents for conidia-forming moulds. Clin. Microbiol. Infect. 14, 982–984. 10.1111/j.1469-0691.2008.02086.x 18828858

[B49] Tampakakis E. Okoli I. Mylonakis E. (2008). A C. *elegans-based, whole animal, in vivo* screen for the identification of antifungal compounds. Nat. Protoc. 3, 1925–1931. 10.1038/nprot.2008.193 19180076

[B50] Tan M. W. Mahajan-Miklos S. Ausubel F. M. (1999). Killing of *Caenorhabditis* elegans by Pseudomonas aeruginosa used to model mammalian bacterial pathogenesis. Proc. Natl. Acad. Sci. U.S.A. 96, 715–720. 10.1073/pnas.96.2.715 9892699 PMC15202

[B51] Tharmalingam N. Rajmuthiah R. Kim W. Fuchs B. B. Jeyamani E. Kelso M. J.. (2018). Antibacterial properties of four novel hit compounds from a methicillin-resistant staphylococcus aureus-caenorhabditis elegans high-throughput screen. Microb. Drug Resist. 24, 666–674. 10.1089/mdr.2017.0250 29461939 PMC6001862

[B52] Thompson T. A. Brown P. D. (2017). Association between the agr locus and the presence of virulence genes and pathogenesis in *Staphylococcus aureus* using a Caenorhabditis elegans model. Int. J. Infect. Dis. 54, 72–76. 10.1016/j.ijid.2016.11.411 27915107

[B53] Vaezi A. Fakhim H. Javidnia J. Khodavaisy S. Abtahian Z. Vojoodi M.. (2018). Pesticide behavior in paddy fields and development of azole-resistant *Aspergillus fumigatus*: should we be concerned? J. Mycol. Med. 28, 59–64. 10.1016/j.mycmed.2017.12.007 29496370

[B54] Vallejo J. A. Beceiro A. Rumbo-Feal S. Rodriguez-Palero M. J. Russo T. A. Bou G. (2015). Optimisation of the *Caenorhabditis* elegans model for studying the pathogenesis of opportunistic *Acinetobacter baumannii*. Int. J. Antimicrob. Agents. S0924-8579(15)00241-1. 10.1016/j.ijantimicag.2015.05.021 26213382

[B55] Van De Veerdonk F. L. Gresnigt M. S. Romani L. Netea M. G. Latgé J.-P. (2017). *Aspergillus fumigatus* morphology and dynamic host interactions. Nat. Rev. Microbiol. 15, 661–674. 10.1038/nrmicro.2017.90 28919635

[B56] Wiederhold N. P. Najvar L. K. Matsumoto S. Bocanegra R. A. Herrera M. L. Wickes B. L.. (2015). Efficacy of the investigational echinocandin ASP9726 in a guinea pig model of invasive pulmonary aspergillosis. Antimicrob. Agents Chemother. 59, 2875–2881. 10.1128/AAC.04857-14 25753643 PMC4394786

[B57] Zaborin A. Romanowski K. Gerdes S. Holbrook C. Lepine F. Long J.. (2009). Red death in *Caenorhabditis* elegans caused by Pseudomonas aeruginosa PAO1. Proc. Natl. Acad. Sci. U.S.A. 106, 6327–6332. 10.1073/pnas.0813199106 19369215 PMC2669342

