# Supplementary material for: Caenorhabditis elegans-Based Aspergillus fumigatus Infection Model for Evaluating Pathogenicity and Drug Efficacy
Source: Front Cell Infect Microbiol. 2020 Jun 26;10:320. doi: 10.3389/fcimb.2020.00320 (PMC7332887; doi:10.3389/fcimb.2020.00320)
Supplement: Supplementary file 1 [file Data_Sheet_1.docx]

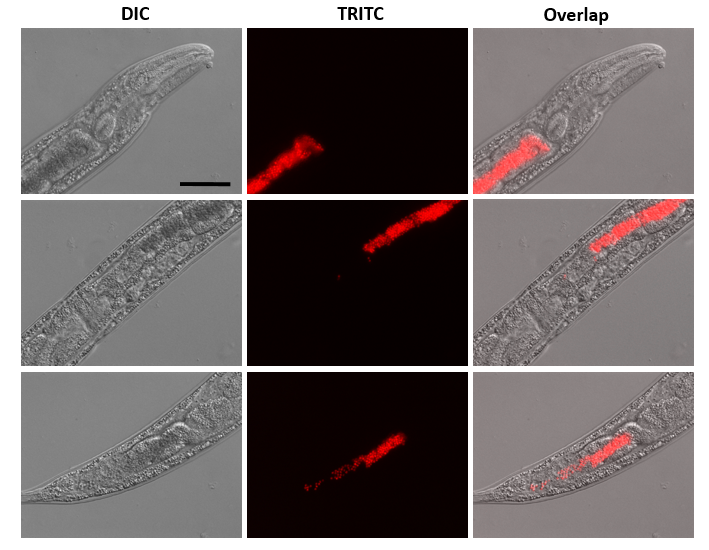


**Supplementary Figure 1.** Infection and progression stages of Af293-dsRed infection to *glp-4*(bn2); *sek-1*(km4) at 0 h in killing assay. Images were taken under DIC and TRITC channels. Scale bar is 50 µm.

**
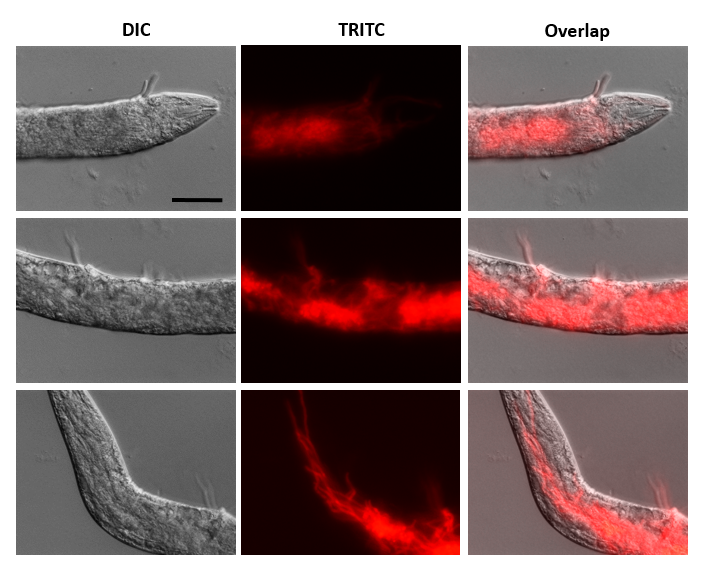
**

**Supplementary Figure 2.** Infection and progression stages of Af293-dsRed infection to *glp-4*(bn2); *sek-1*(km4) at 24 h in killing assay. Images were taken under DIC and TRITC channels. Scale bar is 50 µm.


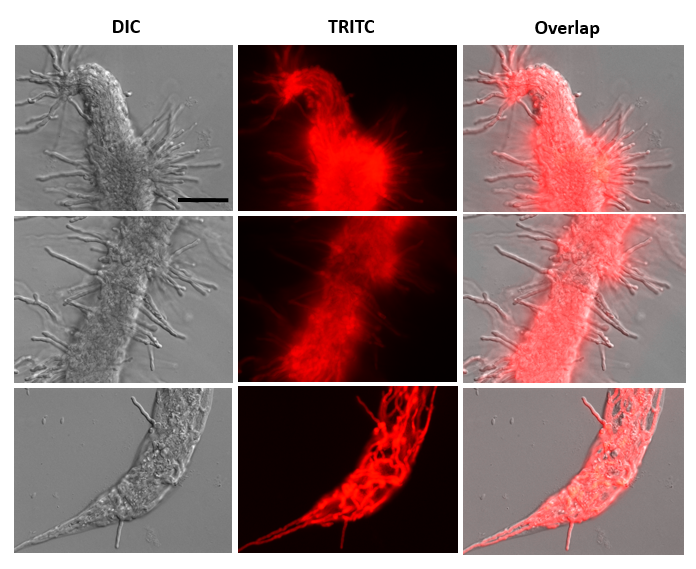


**Supplementary Figure 3.** Infection and progression stages of Af293-dsRed infection to *glp-4*(bn2); *sek-1*(km4) at 48 h in killing assay. Images were taken under DIC and TRITC channels. Scale bar is 50 µm.

**
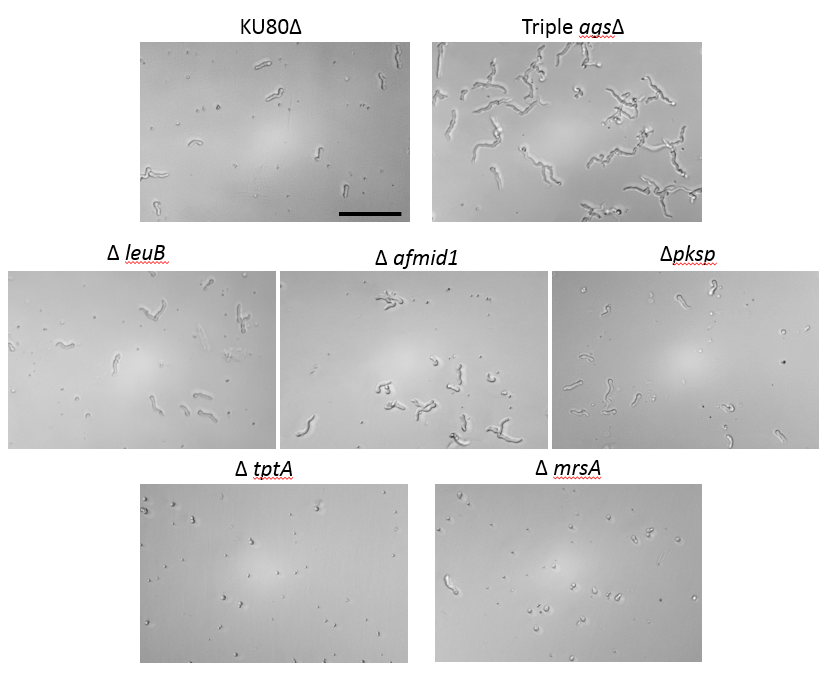
**

**Supplementary Figure 4.** Germination of KU80 and mutant strains in BHI medium for 52 h. Images were taken under DIC channel. Scale bar is 200 µm.

**Supplementary Table 1.** Survival rate of *fem-3*(q96) worms and hyphal filamentation rate from infections by indicated strains. Three biological repeats (each with triplicates) were conducted and total counted worm numbers are indicated.

| Strain | Survival rate (%) | | | % hyphal filamentation at 24 h | Worm numbers |
| --- | --- | --- | --- | --- | --- |
|  | 24 h | 48 h | 72 h |  |  |
| OP50 | 97 ± 2.4 | 96 ± 3.2 | 94 ± 2.5 | 0 | 1748 |
| Af293-dsRed | 96 ± 2.0 | 78 ± 10.0 | 62 ± 8.0 | 5 ± 1.7 | 1111 |
| KU80Δ | 65 ± 6.8 | 37 ± 7.5 | 27 ± 9.0 | 40 ± 6.1 | 920 |
| Dead conidia | 98 ± 2.2 | 96 ± 3.2 | 93 ± 5.2 | 0 | 1647 |

**Supplementary Table 2.** Survival rate of *glp-4*(bn2); *sek-1*(km4) worms and hyphal filamentation rate from infections by indicated strains. Three biological repeats (each with triplicates) were conducted and total counted worm numbers are indicated.

| Strain | Survival rate (%) | | | % hyphal filamentation at 24 h percentage at 24 h | Worm numbers |
| --- | --- | --- | --- | --- | --- |
|  | 24 h | 48 h | 72 h |  |  |
| OP50 | 99 ± 1.5 | 97 ± 2.3 | 94 ± 4.4 | 0 | 2337 |
| Af293 | 80 ± 4.1 | 11 ± 2.1 | 5 ± 2.2 | 46 ± 5.7 | 1119 |
| Af293-dsRed | 81 ± 8.9 | 16 ± 4.2 | 6 ± 1.3 | 36 ± 16 | 1835 |
| KU80Δ | 32 ± 6.4 | 12 ± 3.2 | 8 ± 3.0 | 75 ± 6.4 | 939 |
| Dead conidia | 98 ± 1.2 | 96 ± 2.3 | 91 ± 2.6 | 0 | 1238 |

**Supplementary Table 3.** Survival rate of *glp-4*(bn2); *sek-1*(km4) worms and hyphal filamentation rate from infections by indicated strains. Three biological repeats (each with triplicates) were conducted and total counted worm numbers are indicated.

| Strain | Survival rate (%) | | | % hyphal filamentation at 24 h percentage at 24 h | Worm numbers |
| --- | --- | --- | --- | --- | --- |
|  | 24 h | 48 h | 72 h |  |  |
| KU80Δ | 32 ± 6.4 | 12 ± 3.2 | 8 ± 3.0 | 75 ± 6.4 | 939 |
| Triple *ags*Δ | 88 ± 5.5 | 69 ± 4.2 | 56 ± 6.9 | 16 ± 7.3 | 609 |
| Δ*pksP* | 42 ± 4.8 | 26 ± 4.1 | 20 ± 4.6 | 66 ± 4.2 | 686 |
| Δ*mrsA* | 66 ± 8.6 | 34 ± 8.3 | 19 ± 7.0 | 41 ± 6.9 | 745 |
| Δ*leuB* | 86 ± 5.8 | 54 ± 13.9 | 24 ± 9.1 | 14 ± 4.9 | 566 |
| Δ*tptA* | 88 ± 38 | 49 ± 6.2 | 35 ± 6.9 | 19 ± 5.5 | 1487 |
| *Δafmid1* | 34 ± 4.2 | 13 ± 1.9 | 9 ± 2.5 | 76 ± 6.8 | 1493 |

**Supplementary Table 4.** Survival rate of *glp-4*(bn2); *sek-1*(km4) worms after pre-infection by KU80Δ for 8 h then transferred into liquid killing medium with different concentrations of AmB. Three biological repeats (each with triplicates) were conducted and total counted worm numbers are indicated.

| AmB conc. (µg/ml) | Survival rate (%) | | | Worm numbers |
| --- | --- | --- | --- | --- |
|  | 24 h | 48 h | 72 h |  |
| DMSO | 84 ± 4.8 | 24 ± 11.8 | 10 ± 9.3 | 554 |
| 1.0 | 87 ± 3.1 | 80 ± 4.9 | 72 ± 6.1 | 452 |
| 1.5 | 89 ± 4.2 | 84 ± 4.8 | 81 ± 6.0 | 413 |
| 2.0 | 89 ± 2.6 | 86 ± 3.5 | 83 ± 6.4 | 435 |

**Supplementary Table 5.** Survival rate of *glp-4*(bn2); *sek-1*(km4) worms after pre-infection by KU80Δ for 8 h then transferred into liquid killing medium with different concentrations of ItrZ. Three biological repeats (each with triplicates) were conducted and total counted worm numbers are indicated.

| ItrZ conc. (µg/ml) | Survival rate (%) | | | Worm numbers |
| --- | --- | --- | --- | --- |
|  | 24 h | 48 h | 72 h |  |
| DMSO | 81 ± 8.5 | 27 ± 10.3 | 9 ± 9.7 | 502 |
| 2.0 | 87 ± 6.2 | 64 ± 7.1 | 48 ± 8.0 | 408 |
| 2.5 | 89 ± 5.1 | 70 ± 5.9 | 60 ± 6.6 | 386 |
| 5.0 | 90 ± 5.5 | 80 ± 8.3 | 74 ± 8.1 | 285 |
| 10.0 | 93 ± 5.4 | 87 ± 3.9 | 82 ± 5.9 | 292 |

**Supplementary Table 6.** Survival rate of *glp-4*(bn2); *sek-1*(km4) worms after pre-infection by KU80Δ for 8 h then transferred into liquid killing medium with different concentrations of VoZ. Three biological repeats (each with triplicates) were conducted and total counted worm numbers are indicated.

| VoZ conc. (µg/ml) | Survival rate (%) | | | Worm numbers |
| --- | --- | --- | --- | --- |
|  | 24 h | 48 h | 72 h |  |
| DMSO | 83 ± 8.4 | 19 ± 3.5 | 3.8 ± 1.6 | 430 |
| 0.5 | 91 ± 5.7 | 61 ± 14.2 | 35 ± 9.8 | 390 |
| 1 | 96 ± 3.8 | 85 ± 5.7 | 72 ± 8.4 | 294 |
| 1.5 | 96 ± 4.7 | 90 ± 4.4 | 81 ± 7.2 | 299 |
